# Niche Conservatism Shapes Invasion Patterns of *Opuntia* Species Despite Interspecific Differences in Expansion Dynamics

**DOI:** 10.1002/ece3.74134

**Published:** 2026-08-02

**Authors:** Sifan Tao, Xuefei Chen, Zhechen Qi, Pan Li, Xiaoling Yan

**Affiliations:** ^1^ College of Life Sciences and Medicine Zhejiang Sci‐Tech University Hangzhou China; ^2^ Key Laboratory of National Forestry and Grassland Administration on East China Plant Conservation and Utilization Shanghai Chenshan Botanical Garden Shanghai China; ^3^ Development Center of Plant Germplasm Resources, College of Life Sciences Shanghai Normal University Shanghai China; ^4^ Key Laboratory of Biodiversity and Environment on the Qinghai‐Tibetan Plateau, Ministry of Education, School of Ecology and Environment Xizang University Lhasa China; ^5^ Motuo Biodiversity Observation and Research Station of Xizang Autonomous Region Motuo China

**Keywords:** biological invasion, ecological niche, invasive species dynamics, niche conservatism, niche shift, *Opuntia*

## Abstract

*Opuntia* cacti rank among the most widespread and ecologically damaging plant invaders globally, yet it remains unclear whether their invasion success is driven by niche conservatism or niche shifts. Here, we investigated niche dynamics between native and invaded ranges for four *Opuntia* species ( *O. dillenii* , *O. ficus‐indica* , *O. monacantha* , and *O. humifusa* ) that differ in climatic origin and invasion history. Using biomod2 ensemble models combined with principal component analysis in environmental space (PCA‐env) and multivariate environmental similarity surface (MESS) analysis, we found that all four species exhibit strong niche conservatism, with high niche stability (89.6%–98.2%) and significant niche similarity between native and invaded ranges. Model projections indicated broad climatically suitable areas (10.4–22.5 million km^2^), with invasive occurrences largely confined to native environmental conditions. Niche expansion was limited (1.9%–10.4%) and restricted to marginal environmental conditions. Soil nutrient availability further contributed to distribution patterns in several species, highlighting the role of edaphic factors in shaping niche structure. Despite this overall conservatism, species differed markedly in niche dynamics and geographic expansion, indicating that invasion processes vary substantially among species. Overall, our results suggest that *Opuntia* are primarily characterized by environmental tracking rather than widespread niche shifts, while species‐specific differences in adaptation and spread highlight the importance of assessing invasion risk at the species level.

## Graphical abstract

The graphical abstract illustrates an integrative framework combining ensemble species distribution models, PCA‐env niche quantification, and MESS similarity mapping to assess niche dynamics across four globally invasive *Opuntia* species. Results reveal predominant niche conservatism (89.6%–98.2% stability) with soil nutrient availability identified as a critical distribution driver. These findings support early‐warning surveillance strategies targeting high‐risk regions for invasion.

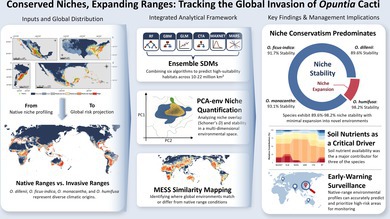

## 1 Introduction

Biological invasions constitute a major component of global change, reshaping biodiversity patterns and ecosystem functioning worldwide (Roy et al. 2024; Simberloff et al. 2013). A central question in invasion ecology concerns the extent to which species retain their ancestral ecological niches when colonizing new regions (Liu et al. 2020). The concept of niche conservatism predicts that species tend to maintain their fundamental environmental requirements across space and time (Wiens et al. 2010; Wiens and Graham 2005), whereas alternative perspectives highlight the potential for niche shifts or expansions under novel environmental conditions (Atwater et al. 2018). Disentangling the relative prevalence of these contrasting outcomes is essential for understanding invasion dynamics and improving predictions of species distributions.

Despite extensive research, empirical evidence for niche conservatism versus niche shift remains inconsistent across taxa (Bates and Bertelsmeier 2021; Peterson 2011). Case studies of widespread invaders illustrate this divergence: species such as *Ambrosia artemisiifolia* exhibit strong niche conservatism (Song et al. 2023), whereas others, including *Lythrum salicaria* , show substantial niche shifts associated with rapid adaptation to novel environments (Colautti and Barrett 2013). However, most studies focus on single species, lacking a comparative framework and thereby making it difficult to disentangle the relative roles of intrinsic ecological constraints and extrinsic factors such as propagule pressure and introduction history (Liu et al. 2020; Rönnfeldt et al. 2026). In addition, comparisons across distantly related taxa may be biased by underlying divergent evolutionary histories (Losos 2008; Münkemüller et al. 2015), constraining inference of general mechanisms (Bates and Bertelsmeier 2021; Diniz‐Filho et al. 2010). A comparative framework within a single lineage, incorporating species with contrasting invasion histories, provides a promising approach to address these limitations.

Implementing this comparative framework requires analytical approaches that integrate both geographic and environmental perspectives. Species distribution models (SDMs) are widely used to predict the potential distributions and identify environmental constraints on species ranges (Elith and Leathwick 2009; Guisan et al. 2013). In parallel, principal component analysis in environmental space (PCA‐env) offers a quantitative framework for assessing niche overlap and characterizing niche dynamics within multidimensional environmental space. By reducing dimensionality and accounting for correlations among environmental variables, PCA‐env offers an integrated representation of realized niches beyond univariate approaches (Liu et al. 2022). Importantly, it allows explicit quantification of niche expansion, stability, and unfilling, enabling standardized comparisons of niche dynamics across ranges (Battini et al. 2019; Guisan et al. 2014). Integrating SDM‐based spatial projections with PCA‐env‐derived niche metrics therefore links geographic patterns with underlying niche processes, providing a robust basis for testing niche conservatism versus niche shift during biological invasions (Guisan et al. 2014).

As one of the most species‐rich genera in Cactaceae, *Opuntia* is widely distributed across the Americas and has emerged as a globally significant group of invaders (Henderson 2007; Walters et al. 2011). Its members possess distinct morphological and physiological traits, including leaves reduced to spines, succulent photosynthetic stems (cladodes), crassulacean acid metabolism (CAM) photosynthesis, and high desiccation tolerance, facilitating establishment across diverse environments (Humphries et al. 2022; Sipango et al. 2022). Owing to their value as livestock fodder and ornamental plants (Inglese et al. 2017; Naorem et al. 2024), *Opuntia* species have been introduced to at least 50 countries worldwide (Githae 2018; Humphries et al. 2022; Novoa et al. 2015; Rasevich et al. 2021; Tesfay and Kreyling 2021). Nearly half of all invasive cacti belong to this genus (Novoa et al. 2015), reflecting both its invasion success and associated socio‐economic impacts (Shackleton et al. 2017; Sipango et al. 2022; Witt et al. 2025). Despite growing attention to *Opuntia* invasions, systematic comparisons of niche dynamics across their global invaded ranges remain limited, particularly regarding whether invaded niches are conserved or shifted relative to native ranges (Bates and Bertelsmeier 2021; Liu et al. 2020; Rönnfeldt et al. 2026).

**FIGURE 1:**
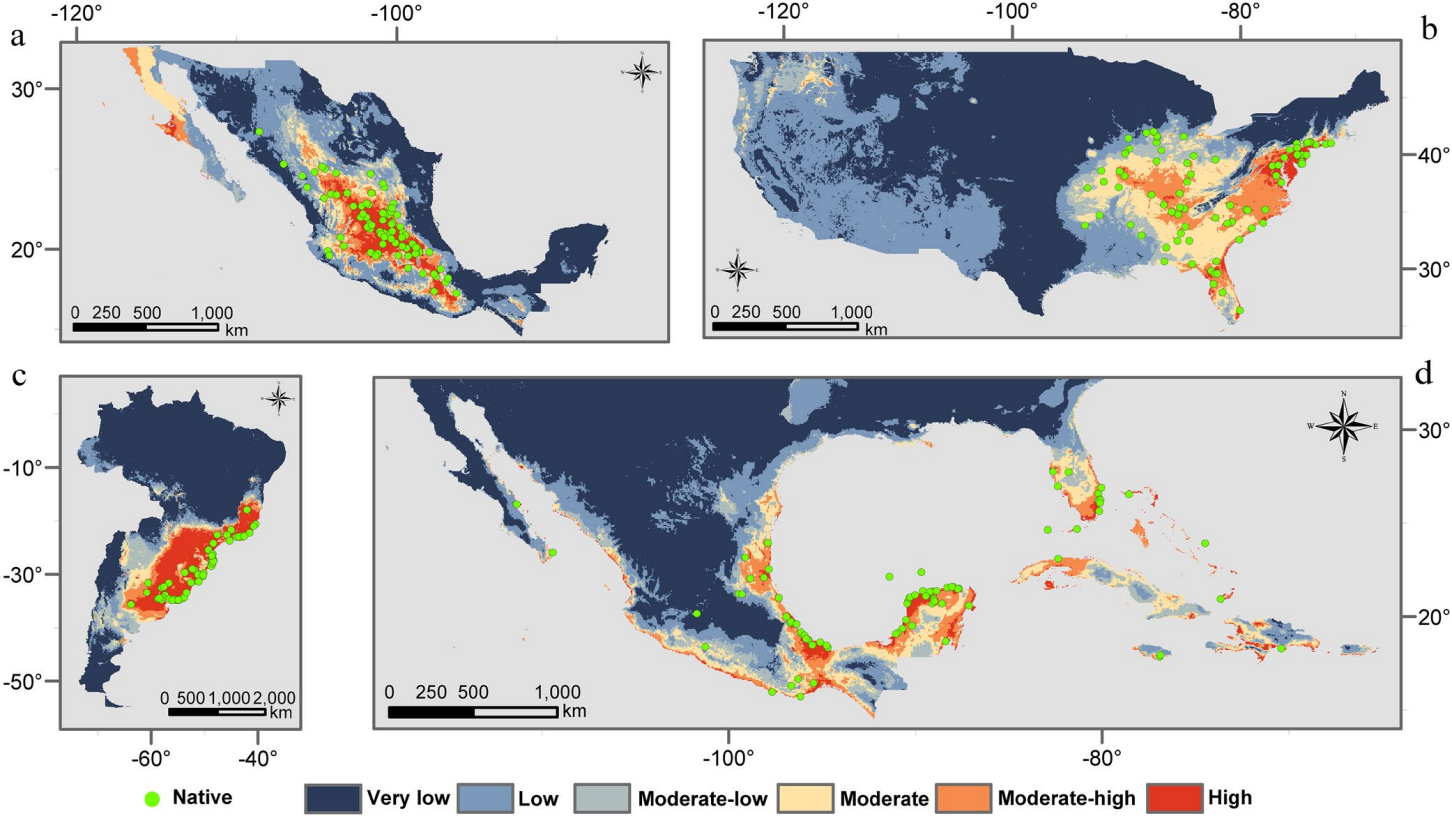
Geographic distribution of native occurrence records for the four *Opuntia* species, with background suitability estimated from native‐range ensemble models to delineate approximate native range boundaries. *O. ficus‐indica* (a), *O. humifusa* (b), *O. monacantha* (c), *O. dillenii* (d).

In this study, we focus on four representative species— *O. ficus‐indica* , *O. dillenii* , *O. monacantha* , and *O. humifusa* —all of which are recognized as significant invaders with documented established populations across Europe, Africa, and Asia (Baumgartner et al. 2025; EPPO 2026; GISD 2026; Yan et al. 2024). These species span a broad climatic gradient in their native ranges across the Americas, from tropical to temperate environments (Abay 2018; Goldstein and Nobel 1994; Sakhraoui et al. 2022) (Figure 1). In addition to climatic variation, they differ markedly in invasion histories and residence times. *Opuntia ficus‐indica* has a long history of human‐mediated introduction and cultivation dating back to the late 15th century, resulting in high propagule pressure and widespread global distribution (Griffith 2004; Imms 1941; Kiesling 1998). *O. dillenii* has also been widely introduced, particularly in tropical regions, and is well established in many coastal and island systems (Shackleton et al. 2017). By contrast, *O. monacantha* and *O. humifusa* remain more restricted in their invaded ranges, likely reflecting shorter residence times and earlier invasion stages rather than intrinsic differences in ecological tolerance. Together, this combination of climatic diversity and contrasting invasion histories provides a robust comparative framework to disentangle the relative roles of environmental constraints and dispersal processes in shaping niche dynamics (Carscadden et al. 2020; Liu et al. 2022).

Using this comparative approach, we test whether niche dynamics in *Opuntia* invasions are primarily governed by niche conservatism or involve detectable niche shifts. Specifically, we: (1) project potential distributions and range expansion using ensemble SDMs; (2) quantify niche overlap, expansion, stability, and unfilling by comparing native and invasive occurrence in environmental space (PCA‐env); and (3) identify key environmental drivers using variable importance metrics and multivariate environmental similarity surface (MESS) mapping. Together, these approaches provide a comprehensive basis for evaluating niche dynamics during *Opuntia* invasions and improve the prediction of invasion risk in vulnerable regions.

## 2 Materials and Methods

### 2.1 Occurrence Data Collection and Preprocessing

Occurrence records for the four target *Opuntia* species ( *O. dillenii* , *O. ficus‐indica* , *O. monacantha* , and *O. humifusa* ) were retrieved from the Global Biodiversity Information Facility (GBIF, https://www.gbif.org/). To ensure comprehensive data collection, we selected the accepted species name for each taxon so that records filed under synonyms were consolidated through the GBIF taxonomic backbone. Because authoritative checklists differ in their treatment of *O. dillenii* , we followed the Flora of North America (Pinkava 2003), which recognizes it as a distinct species, and verified accepted names and synonymies accordingly. Native ranges were defined based on published literature, regional floras, and curated online databases: *O. dillenii* —southeastern United States and Caribbean coast (Bernal et al. 2016; Negrão et al. 2022; Pickering and Awale 2018; Vázquez Pardo and García Alonso 2017); *O. humifusa* —eastern United States (Pinkava 2003; U.S. Forest Service 2026; Yan et al. 2024); *O. monacantha* —eastern South America (Brazil, Uruguay, Paraguay, and Argentina) (Lim 2012; Novoa et al. 2015) (Figure 1); *O. ficus‐indica* —central Mexico (Griffith 2004; Reyes‐Agüero et al. 2005). Recent phylogenetic studies indicate that *O. ficus‐indica* represents a domestication complex comprising multiple closely related lineages originating from central Mexico (Griffith 2004). To better characterize its native niche, we therefore included occurrence records of five closely related taxa ( *O. megacantha* , *O. streptacantha* , *O. tomentosa* , *O. leucotricha* , and *O. hyptiacantha* ), which have narrower distributions and are far less affected by human translocation than cultivated lineages.

Data cleaning and standardization were performed in R (v4.5.2; R Core Team 2025), using dplyr (v1.1.4; Wickham et al. 2023) for data manipulation, sf (v1.1.0; Pebesma 2018) and rnaturalearth (v1.1.0; Massicotte and South 2025) for spatial processing, and spThin (v0.2.0; Aiello‐Lammens et al. 2015) for spatial thinning. The following procedures were applied: (1) removing records lacking geographic coordinates or with zero values; (2) excluding entries with “cultivated”, “market”, “zoo” and “garden” keywords; (3) discarding points with coordinate uncertainty > 10 km; (4) eliminating marine occurrences via terrestrial boundary verification; (5) applying spatial thinning to one record per 5 km × 5 km grid to reduce sampling bias and spatial autocorrelation (Li et al. 2025). The final datasets comprised: *O. humifusa* (native: 1181; invasive: 1152), *O. dillenii* (native: 171; invasive: 837), *O. monacantha* (native: 136; invasive: 1067), and *O. ficus‐indica* (native: 1227; invasive: 8271) (Table S1).

### 2.2 Environmental Variable Collection and Selection

Bioclimatic variables, together with solar radiation and soil variables, were used to characterize the environmental conditions influencing the distribution of *Opuntia* species, given the importance of radiation for CAM photosynthetic efficiency and soil properties for root function in cacti (Nobel 2002; Novoa et al. 2015). Bioclimatic variables and monthly solar radiation data were obtained from the WorldClim database (v2.1, https://www.worldclim.org/) at a spatial resolution of 2.5 arc‐minutes; soil variables (seven in total) were obtained from the Harmonized World Soil Database (HWSD v1.2, https://gaez.fao.org/) and resampled to the same resolution using the nearest neighbor interpolation (Masocha and Dube 2018) (Table S2). To reduce redundancy among correlated predictors and improve the interpretability of the variable‐importance estimates we report, a two‐step variable selection procedure was implemented in R (Díaz‐Vallejo et al. 2024). First, Pearson correlation coefficients were calculated to assess pairwise relationships among variables. Second, the variance inflation factor (VIF) was used to identify redundant predictors. Variables were retained using thresholds of |*r*| < 0.6 and VIF < 3 (Celemín et al. 2025; Krah et al. 2019), resulting in a final set of eleven predictors for subsequent modeling (Table 1).

**TABLE 1:** Environmental variables retained after variable selection for species distribution modeling.

Category	Variable	Description
Temperature	bio2	Mean diurnal range
	bio5	Maximum temperature of warmest month
	bio8	Mean temperature of wettest quarter
	bio9	Mean temperature of driest quarter
Precipitation	bio13	Precipitation of wettest month
	bio19	Precipitation of coldest quarter
Solar radiation	srad04	April solar radiation
	srad09	September solar radiation
Soil	sq1	Nutrient availability
	sq3	Rooting conditions
	sq4	Oxygen availability

### 2.3 Species Distribution Modeling

We implemented a multimodel framework in the biomod2 (v4.3–4‐3; Guéguen et al. 2025) combining multiple algorithms, multimetric evaluation, and ensemble modeling to improve the robustness and predictive performance of species distribution models for the four *Opuntia* species. Specifically, ten algorithms available in biomod2 were initially evaluated. For each species and dataset, 5000 pseudo‐absence points were generated using the uniform random sampling strategy (Barbet‐Massin et al. 2012). For native‐range models, the study area was defined as the intersection of each species' documented native range with the bounding box of its native occurrences; for global models, the study area comprised the global terrestrial extent. Models were calibrated and evaluated using a five‐fold cross‐validation procedure, with 75% of occurrence records used for training and 25% for testing in each run (Xian et al. 2023). Model performance was assessed using the area under the receiver operating characteristic curve (AUC), the true skill statistic (TSS), and Cohen's Kappa (Allouche et al. 2006; Pearce and Ferrier 2000). Artificial Neural Networks (ANN) were excluded due to frequent convergence failures and unstable predictions across runs. To ensure robust model performance, only models achieving a mean TSS > 0.7 were retained for further ensemble modeling (Meller et al. 2014). Based on this criterion, six algorithms were selected: Maximum Entropy (MAXNET), Generalized Linear Model (GLM), Multivariate Adaptive Regression Splines (MARS), Generalized Boosting Model (GBM), Classification Tree Analysis (CTA), and Random Forest (RF).

The selected models were integrated into an ensemble model using a weighted mean approach, where model weights were proportional to their TSS scores, as implemented in biomod2. Model parameterization remained consistent with the selection phase to ensure methodological continuity. The final spatial outputs were imported into ArcGIS (v10.8; Esri 2022) for further processing. For spatial quantification, continuous suitability predictions (0–1) were converted to binary maps using the maximum TSS threshold, which optimally balances sensitivity and specificity (Liu et al. 2013). This enabled comparison of suitable habitat areas between native and global datasets. To identify ecological drivers, variable importance was quantified using permutation analysis based on the global occurrence dataset, with TSS reductions calculated after 10 randomizations per variable. These values were then standardized to relative contributions (%) and used to assess the overall importance of each predictor.

### 2.4 Niche Quantification and Dynamics Analysis

Niche dynamics were quantified using the PCA‐env framework implemented in the ecospat package (v4.1.3; Broennimann et al. 2025). Because the species' seeds are dispersed over relevant ecological timescales through animal‐mediated movement (Humphries et al. 2022), M was operationally delineated as a 100‐km buffer around occurrence records; this buffer width is consistent with values commonly used to approximate accessible areas in niche modeling (Anderson and Raza 2010; Barve et al. 2011; Cooper and Soberón 2018). Background points were sampled within each species' M and restricted to terrestrial areas using a land mask (VanDerWal et al. 2009). A global environmental space was constructed by pooling environmental conditions from background points of all four species, providing a common environmental reference for cross‐species comparison. Principal component analysis (PCA) was performed on the eleven selected variables after z‐transformation. Environmental variables were incorporated to represent a multidimensional environmental space consistent with the SDM framework. The PCA space was discretized into a 100 × 100 grid, and kernel density smoothing was applied to estimate occurrence densities relative to background environmental availability (Broennimann et al. 2012). For visualization, niche occupancy was delineated by retaining grid cells where kernel density exceeded 5% of the species‐specific maximum density, following the approach of Broennimann et al. (2012). Regions exclusive to the native range, exclusive to the invasive range, and shared between both ranges are displayed separately to facilitate visual assessment of niche overlap. Occurrence points were randomly thinned for display clarity.

Niche overlap between native and invaded ranges was quantified using Schoener's *D*, ranging from 0 (no overlap) to 1 (complete overlap) (Warren et al. 2008). A niche similarity test with 1000 permutations (*α* = 0.05) was conducted to assess whether observed overlap exceeded random expectations. Following Broennimann et al. (2012), niche dynamics were decomposed into three components: stability (shared environmental space), expansion (environmental space occupied only in the invaded range), and unfilling (native environmental space not occupied in the invaded range), providing a quantitative assessment of niche conservatism and divergence during invasion.

### 2.5 Multivariate Environmental Similarity Surface (MESS) and Most Dissimilar Variable (MoD) Analyses

Multivariate environmental similarity surface (MESS) analysis was used to characterize environmental similarity between native and invaded ranges and to identify limiting environmental conditions across geographic space (Elith et al. 2010). Prior to MESS computation, the upper and lower 0.5% of native reference values for each environmental variable were trimmed to minimize the influence of outliers. For each environmental variable *V*_*i*_ at a given location, similarity was calculated relative to the native environmental reference as follows: when a value *p* fell within the native range, similarity corresponded to the percentile position of *p* within the reference distribution; when *p* lay outside this range, similarity was quantified as the normalized distance to the nearest boundary (Elith et al. 2010). The overall MESS value at each location was defined as the minimum similarity across all variables, thereby indicating the most limiting environmental factor. MESS values are centered around zero, with positive values indicating environmental conditions fully represented within the native range and negative values indicating conditions outside the native environmental envelope for at least one variable (Elith et al. 2010).

To further identify the environmental variables contributing most strongly to environmental dissimilarity, we conducted a Most Dissimilar Variable (MoD) analysis (Elith et al. 2010). This approach identifies, for each location, the variable responsible for the lowest MESS value. MESS and MoD outputs were processed and visualized in ArcGIS 10.8 (Esri 2022).

## 3 Results

### 3.1 Model Performance and Potential Distribution Patterns

**FIGURE 2:**
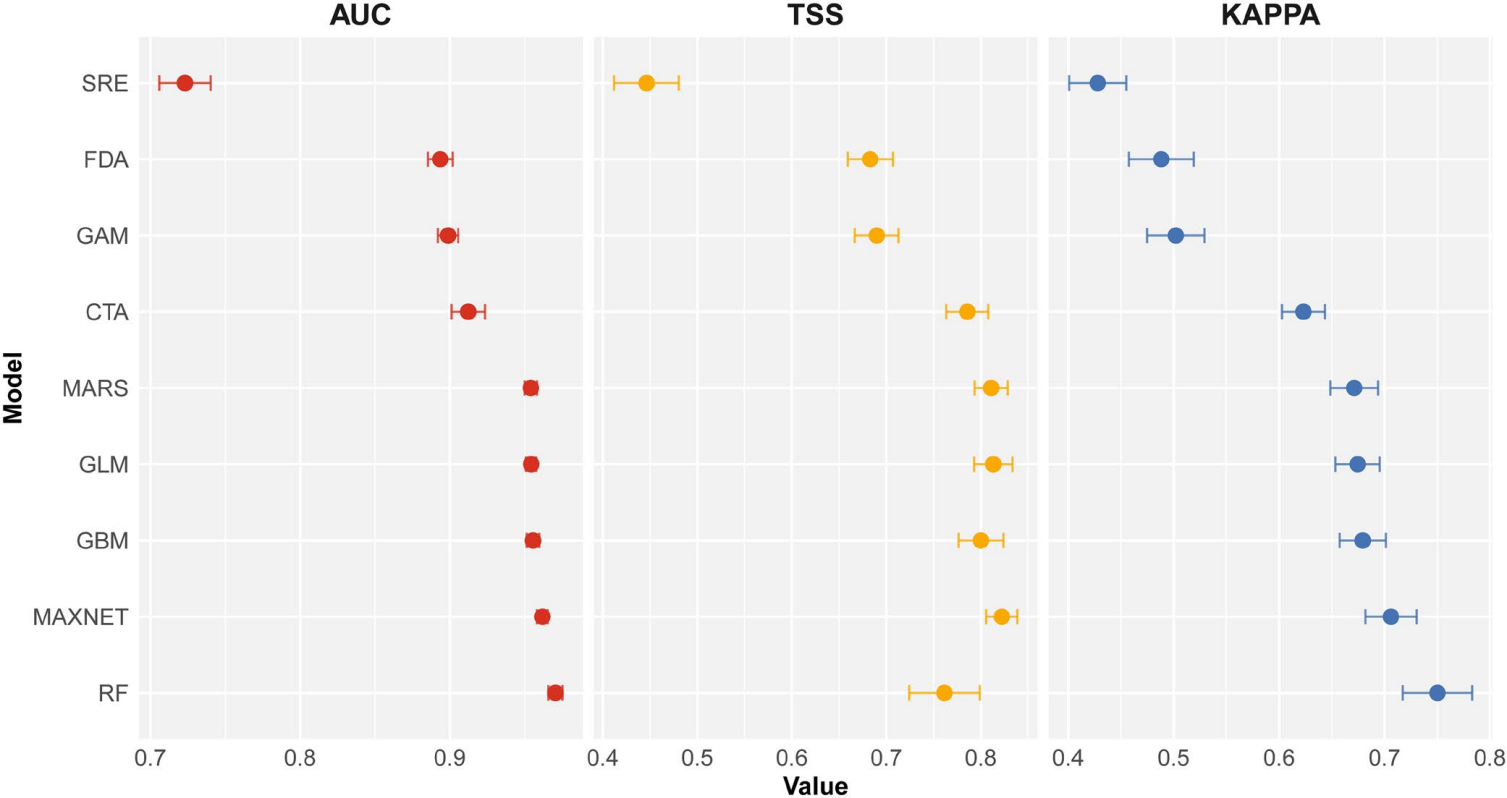
Comparison of predictive performance across nine modeling algorithms based on the area under the receiver operating characteristic curve (AUC), the true skill statistic (TSS), and Cohen's Kappa. Models with mean TSS > 0.7 were retained for ensemble modeling. Points represent mean values, and error bars indicate variability across model runs.

A total of ten modeling algorithms were initially evaluated (Table S3). Based on model performance, algorithms with a mean TSS > 0.7 were retained for ensemble modeling, resulting in six selected models (CTA, GBM, GLM, MARS, MAXNET, RF) (Figure 2). Among the retained models, RF and MAXNET demonstrated the highest predictive performance. RF achieved mean values of AUC, TSS, and Kappa of 0.971, 0.761, and 0.75, respectively, while MAXNET achieved corresponding values of 0.962, 0.822, and 0.706.

**FIGURE 3:**
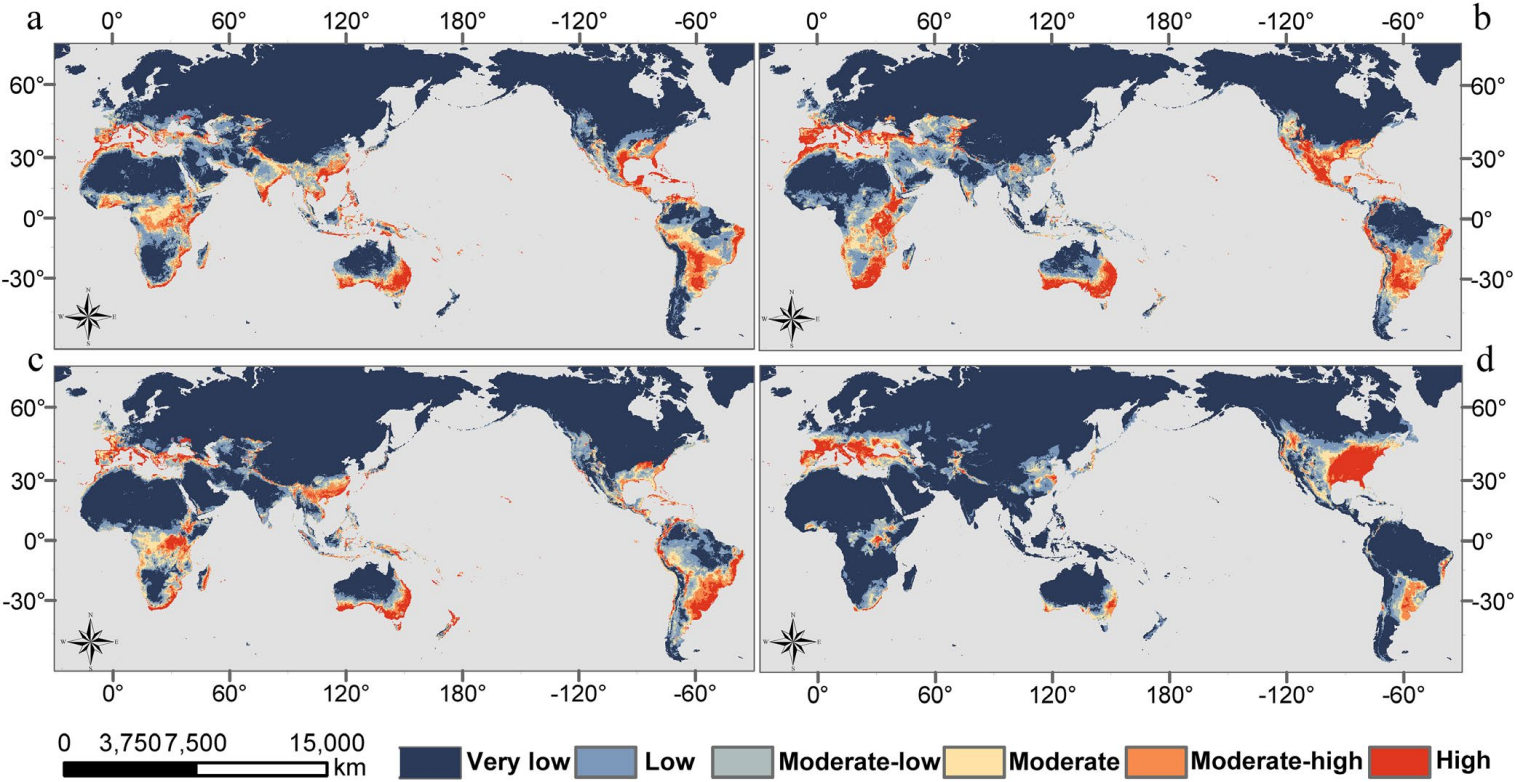
Predicted global potential distribution of four *Opuntia* species under current climatic conditions: (a) *O. dillenii* , (b) *O. ficus‐indica* , (c) *O. monacantha* , and (d) *O. humifusa* . Suitability classes are shown from very low to high based on ensemble model predictions.

The predicted suitable habitats of the four *Opuntia* species exhibited distinct yet partially overlapping global patterns across tropical, subtropical, and temperate regions (Figure 3). These potential habitats are primarily concentrated in Mediterranean climates and subtropical monsoon regions. Species‐specific distributions differed markedly. *O. dillenii* showed high suitability in tropical and subtropical coastal regions, particularly in the Caribbean, Central America, northern South America, and parts of Southeast Asia and northern Australia (Figure 3a). *O. ficus‐indica* exhibited the broadest potential distribution, with extensive suitable areas across subtropical and warm temperate regions, including the Mediterranean Basin, East Africa, South Asia, South America, and Australia (Figure 3b). *O. monacantha* was mainly distributed in the Southern Hemisphere, with suitable habitats in South America, southern Africa, and Australia, and limited presence in the Northern Hemisphere (Figure 3c). In contrast, *O. humifusa* was largely restricted to temperate regions of the Northern Hemisphere, with high suitability in eastern North America and additional areas in Europe and East Asia (Figure 3d).

Binary habitat analysis revealed that, for all four *Opuntia* species, the predicted suitable area at the global scale substantially exceeded that within their native ranges (Table 2). Global suitable areas ranged from 10.4 to 22.5 million km^2^, indicating widespread environmental availability beyond their regions of origin. Among the species, *O. humifusa* exhibited the smallest global suitable area (10,413,377 km^2^), whereas *O. dillenii* , *O. ficus‐indica* , and *O. monacantha* showed considerably larger extents (17–22 million km^2^). Expansion ratios varied markedly among species, ranging from 10.0 ( *O. monacantha* ) to 46.2 ( *O. ficus‐indica* ), with *O. dillenii* also exhibiting a high expansion ratio (43.5).

**TABLE 2:** Predicted suitable habitat areas and expansion ratios for four *Opuntia* species under native and global scenarios.

Species	Native suitable area (km^2^)	Global suitable area (km^2^)	Expansion ratio
*O. dillenii*	516,849	22,466,488	43.5
*O. ficus‐indica*	381,634	17,633,562	46.2
*O. humifusa*	718,164	10,413,377	14.5
*O. monacantha*	1,740,668	17,477,450	10.0

### 3.2 Environmental Determinants of Species Distributions

**FIGURE 4:**
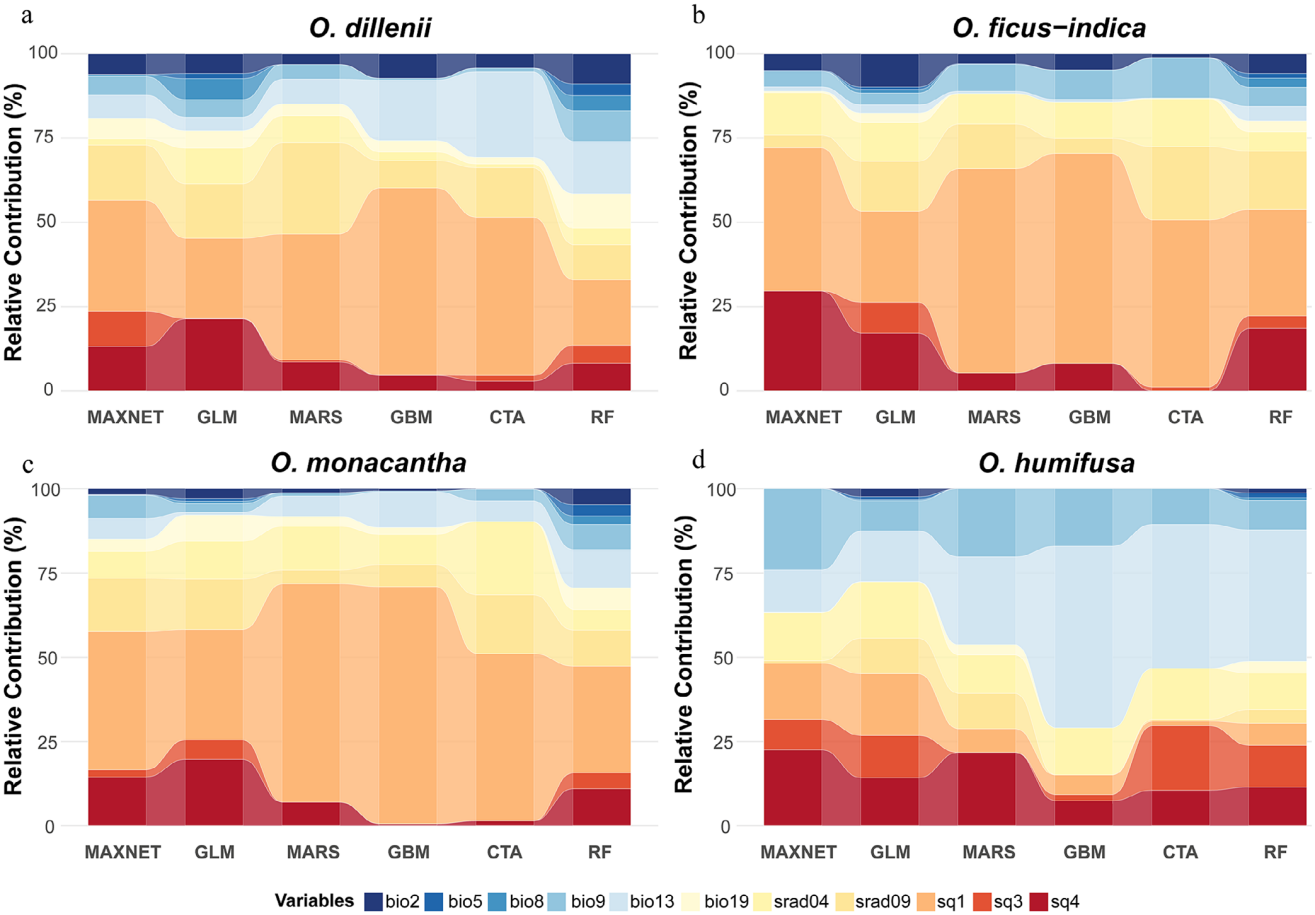
Relative contributions (%) of environmental variables across different modeling algorithms for each *Opuntia* species. Stacked bar plots show the proportional contribution of each predictor variable within individual models. Panels represent (a) *O. dillenii* , (b) *O. ficus‐indica* , (c) *O. monacantha* , and (d) *O. humifusa* .

Permutation‐based variable contribution analysis revealed clear interspecific differences in environmental predictors (Figure 4). Across modeling algorithms, variable importance showed broadly consistent patterns, with structured variation among algorithms (Figure 4). In particular, similar contribution structures were evident within two groups of models: MAXNET, GLM, and RF showed broadly consistent patterns, while MARS, GBM, and CTA also exhibited internally consistent but distinct contribution structures. Soil‐related variables generally played a dominant role in shaping model predictions for *O. monacantha* , *O. ficus‐indica* , and *O. dillenii* . Soil nutrient availability (sq1) contributed the largest proportion of explained variation in these species, accounting for 48.4%, 45.6%, and 36.0%, respectively (Table 3). In addition, oxygen availability to roots (sq4) also contributed substantially across models (Figure 4). Notably, sq4 ranked as the second most important predictor for *O. ficus‐indica* and the third for *O. humifusa* (Table 3).

**TABLE 3:** Top three environmental predictors and their relative contribution for each species.

Species	1st (Var/%)	2nd (Var/%)	3rd (Var/%)
*O. dillenii*	sq1 (36.0)	srad09 (15.5)	bio13 (12.9)
*O. ficus‐indica*	sq1 (45.6)	sq4 (13.1)	srad09 (12.6)
*O. humifusa*	bio13 (31.6)	bio9 (14.9)	sq4 (14.7)
*O. monacantha*	sq1 (48.4)	srad09 (11.6)	srad04 (11.5)

Solar radiation variables also contributed to model performance. September solar radiation (srad09) consistently ranked among the top three predictors for *O. monacantha* (11.6%), *O. ficus‐indica* (12.6%), and *O. dillenii* (15.5%), while April solar radiation (srad04) ranked as the third most important predictor for *O. monacantha* (11.5%), although its relative contributions varied among models.

By contrast, *O. humifusa* exhibited a different contribution pattern, with precipitation and temperature variables dominating across models. Precipitation of the wettest month (bio13) was the primary predictor (31.6%), followed by mean temperature of the driest quarter (bio9; 14.9%) and oxygen availability to roots (sq4; 14.7%).

### 3.3 Niche Dynamic Analysis

**FIGURE 5:**
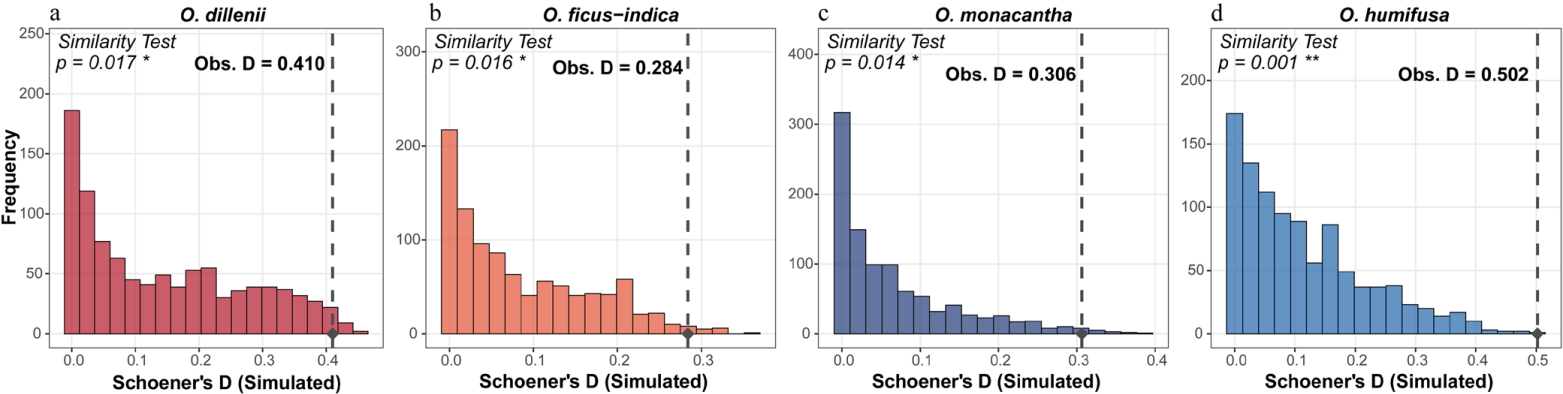
Results of niche similarity tests based on Schoener's *D* for four *Opuntia* species. Histograms represent the null distributions of Schoener's *D* generated from 1000 random permutations under the niche similarity test framework. The vertical dashed line indicates the observed Schoener's *D* value for each species pair. *p*‐values denote the proportion of simulated values exceeding the observed value. Panels correspond to (a) *O. dillenii* , (b) *O. ficus‐indica* , (c) *O. monacantha* , and (d) *O. humifusa* .

PCA‐env analysis, conducted within a shared environmental space where PC1 and PC2 explained 47.9% of the total variance (29.50% and 18.43%, respectively), was used to quantify niche overlap and dynamics between native and invaded ranges. The contributions of individual environmental variables to these axes are summarized in the principal component loadings (Table S4). Schoener's *D* values ranged from 0.284 to 0.502, indicating moderate levels of niche overlap across species. The highest overlap was observed in *O. humifusa* (0.502), followed by *O. dillenii* (0.410), *O. monacantha* (0.306), and *O. ficus‐indica* (0.284) (Table 4). One‐tailed similarity tests were significant for all species (*p* < 0.05), demonstrating that the observed niche overlap exceeded expectations under a random environmental allocation scenario (Figure 5).

**TABLE 4:** Niche overlap (Schoener's *D*) and niche dynamics components (expansion, stability, and unfilling) for four *Opuntia* species.

Species	Schoener's *D*	Expansion	Stability	Unfilling
*O. ficus‐indica*	0.2840	0.0830	0.9170	0.0000
*O. dillenii*	0.4100	0.1036	0.8964	0.0072
*O. monacantha*	0.3062	0.0693	0.9307	0.0003
*O. humifusa*	0.5021	0.0185	0.9815	0.0000

Niche dynamics decomposition provided further insights into these patterns. Stability values were consistently high across species, ranging from 89.6% to 98.2%, indicating that invasive populations predominantly occupied climatic conditions already represented within their native niches. *O. dillenii* exhibited the highest proportional expansion (10.4%), followed by *O. ficus‐indica* (8.3%) and *O. monacantha* (6.9%). In contrast, *O. humifusa* showed the lowest expansion (1.9%) and the highest stability (98.2%).

**FIGURE 6:**
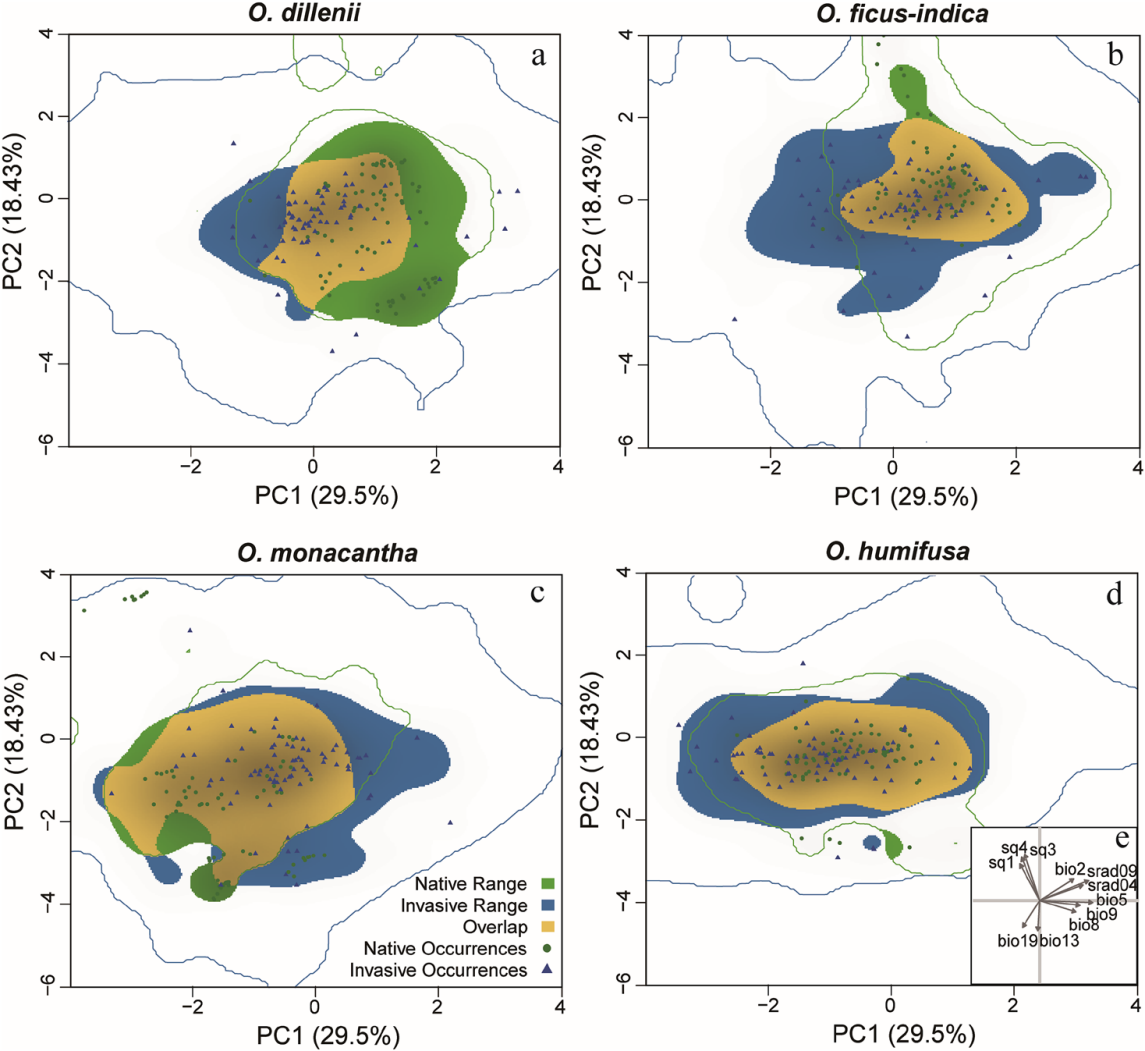
Niche dynamics of four *Opuntia* species in PCA‐defined environmental space based on the PCA‐env approach. Niche changes are decomposed into stability (green), expansion (blue), and unfilling (yellow). Contour lines indicate the extent of environmental space, and arrows show the contribution of environmental variables to the principal components (e). Panels represent (a) *O. dillenii* , (b) *O. ficus‐indica* , (c) *O. monacantha* , and (d) *O. humifusa* .

Each species displayed a distinct spatial pattern of expansion in environmental space (Figure 6, Table 4). *O. dillenii* exhibited the largest proportional expansion (expansion = 0.104) among the four species, with its native niche occupying the positive PC1 region associated with higher temperature and solar radiation loadings; expansion occurred predominantly along the negative PC1 direction (Figure 6a). *O. ficus‐indica* exhibited relatively large niche expansion (expansion = 0.083), with regions extending along both the negative PC1 and negative PC2 axes, toward the direction of bio19 (Figure 6b). *O. monacantha* showed expansion predominantly toward the positive PC1 direction (Figure 6c). In contrast, *O. humifusa* exhibited the narrowest native niche breadth and the highest niche stability (stability = 0.982, expansion = 0.019), with only limited expansion along both directions of PC1 (Figure 6d).

### 3.4 Environmental Similarity Patterns and Key Limiting Variables

**FIGURE 7:**
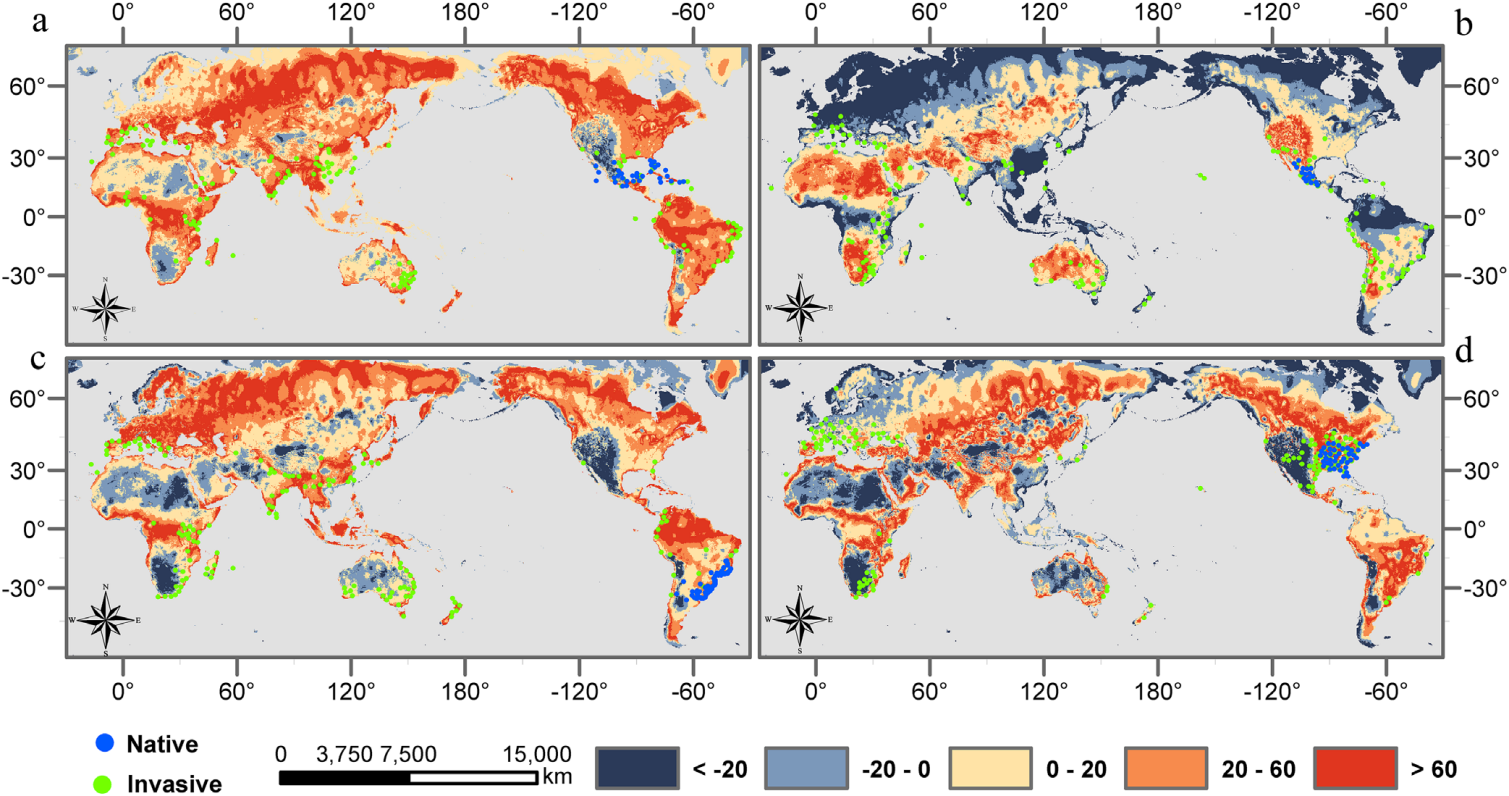
Multivariate environment similarity surfaces (MESS) for four *Opuntia* species. MESS values quantify the similarity between environmental conditions in the invaded range and those in the native range. Positive values (MESS > 0; warm colors) indicate environments analogous to the native range, whereas negative values (MESS < 0; cool colors) represent novel environmental conditions outside the native niche. The magnitude of negative values reflects the degree of environmental dissimilarity. Panels correspond to (a) *O. dillenii* , (b) *O. ficus‐indica* , (c) *O. monacantha* , and (d) *O. humifusa* .

**FIGURE 8:**
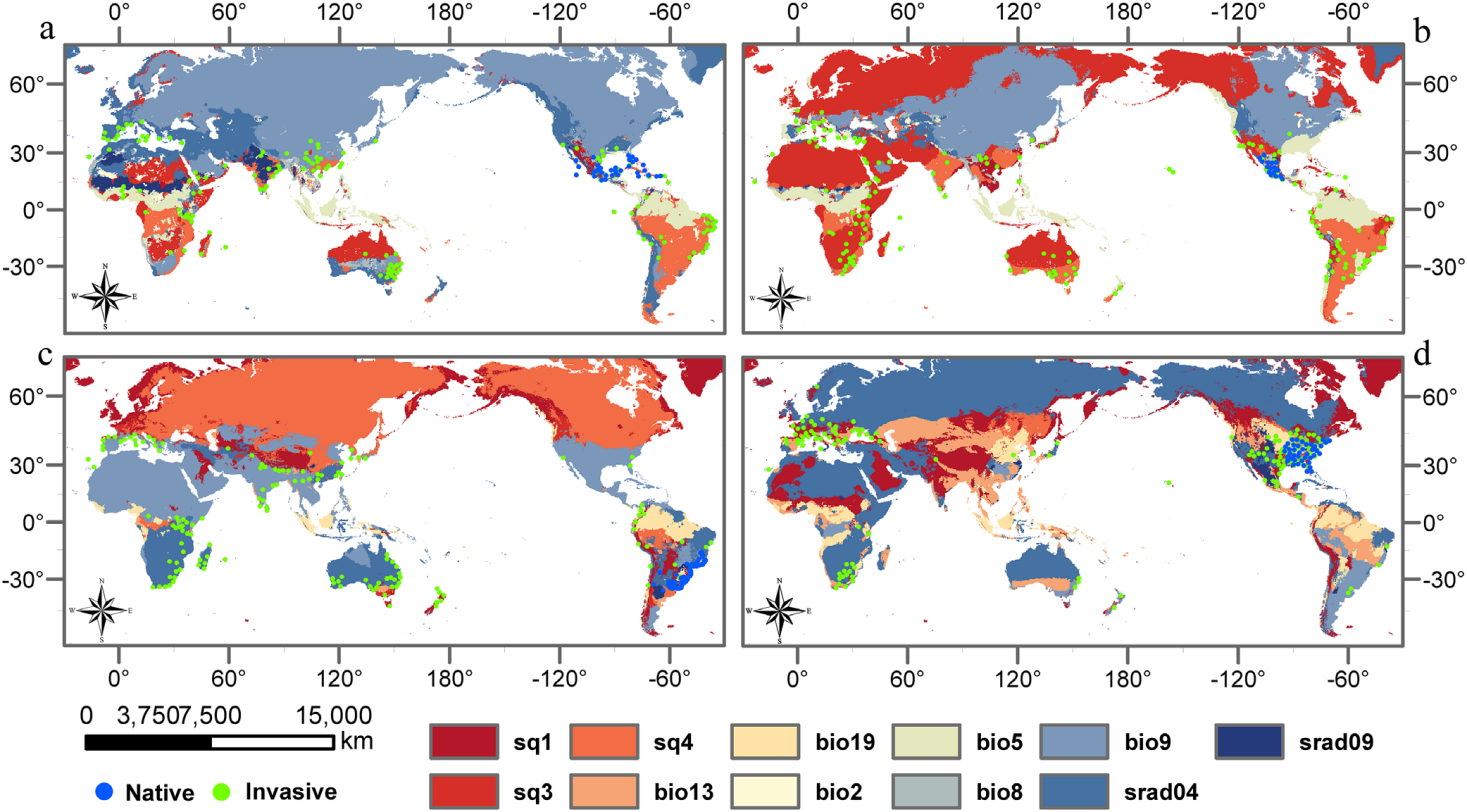
Spatial distribution of the most dissimilar environmental variables (MoD) for four *Opuntia* species. MoD maps identify the variable responsible for the lowest similarity (i.e., greatest environmental dissimilarity) between invaded and native ranges at each location, derived from the MESS analysis. Each color represents the environmental predictor contributing most strongly to environmental novelty. Panels correspond to (a) *O. dillenii* , (b) *O. ficus‐indica* , (c) *O. monacantha* , and (d) *O. humifusa* .

We mapped the global environmental correspondence between native and invaded ranges (Figure 7) and identified the primary drivers of regional dissimilarity (Figure 8). Most invasive occurrences across all species were located in areas with positive MESS values, indicating that environmental conditions largely fall within the native climatic and soil‐related niche space. Occurrences in environmentally dissimilar areas (MESS < 0) were relatively rare and varied among species. *O. ficus‐indica* exhibited the highest number of such occurrences, distributed across South America, the Mediterranean, and East Asia, and associated with both climatic and soil‐related variables (e.g., bio5, sq3, sq4, bio9). *O. humifusa* showed several occurrences in dissimilar environments, primarily in southern Africa and southwestern North America, associated with solar radiation and soil‐related variables, whereas *O. dillenii* and *O. monacantha* exhibited minimal presence in environmentally novel conditions. Overall, most invasive occurrences were located in environmentally similar areas, with only a limited proportion occurring in dissimilar conditions.

## 4 Discussion

### 4.1 Niche Conservatism With Limited Expansion in Opuntia Invasion

Our results illustrate a predominant role of niche conservatism in *Opuntia* invasions, with species largely occupying environmental conditions analogous to those in their native ranges. This pattern is evidenced by consistently high niche stability (> 89%) and minimal niche unfilling (Table 4), together with the concentration of invasive occurrences in environmentally analogous regions (Figure 7). Rather than indicating fundamental niche divergence (Broennimann et al. 2012), the relatively low to moderate Schoener's *D* values (0.284–0.502) likely reflect differences in occurrence density within a broadly conserved environmental space, as also apparent in the PCA projections (Figure 6). Despite this overall conservatism, limited niche expansion (1.9%–10.4%) was observed across species, indicating that invasion may involve the occupation of peripheral environmental conditions adjacent to the native niche (Guisan et al. 2014; Qiao et al. 2017). Such expansion is likely constrained by underlying physiological tolerances, suggesting that realized niche shifts occur primarily within, rather than beyond, the boundaries of the fundamental niche (Liu et al. 2020; Tingley et al. 2014).

These findings are consistent with a growing body of evidence indicating that climatic niche conservatism predominates in plant invasions. For example, invasive populations of *Ambrosia artemisiifolia* and *Solidago canadensis* exhibit extensive geographic expansion while largely retaining their native climatic niches, with only limited expansion at environmental margins (Petitpierre et al. 2012; Song et al. 2023). Similarly, tropical invaders such as *Tithonia diversifolia* and *Chromolaena odorata* show high niche stability and strong climatic overlap between native and invaded ranges (Obiakara and Fourcade 2018; Zhang et al. 2023), suggesting that invasion success is primarily driven by dispersal into preadapted environments rather than rapid niche evolution (Petitpierre et al. 2012; Wiens et al. 2010). In cacti, particularly within *Opuntia*, global invasion patterns are likewise largely confined to arid and semiarid climatic envelopes, further supporting the predominance of niche conservatism (Novoa et al. 2015; Novoa, Brundu, et al. 2019; Shackleton et al. 2017).

More broadly, these results reinforce the view that biological invasions are typically governed by niche conservatism at macroecological scales, while allowing for limited niche expansion within existing physiological constraints (Atwater et al. 2018; Guisan et al. 2014; Petitpierre et al. 2012). In this context, *Opuntia* invasion appears to reflect environmental tracking across geographically distinct but climatically analogous regions, rather than adaptive shifts into fundamentally novel environmental space.

### 4.2 Soil‐Related Factors as an Important Dimension of Opuntia Niche Structure

While climatic variables capture the primary axis of niche conservatism, soil‐related factors further refine the ecological niche structure of *Opuntia* species. In particular, soil nutrient availability (sq1) consistently ranked as a dominant predictor for *O. monacantha* (48.4%), *O. ficus‐indica* (45.6%), and *O. dillenii* (36.0%), exceeding the contribution of most climatic variables. This pattern highlights the importance of substrate conditions as a key, yet often underrepresented, dimension of niche differentiation, consistent with recent macroecological findings in Cactaceae (Thompson et al. 2024). Although *Opuntia* species are generally adapted to arid and nutrient‐poor environments, physiological evidence indicates that their growth and establishment remain sensitive to soil properties, including nutrient availability, texture, and pH (Inglese et al. 2017; Nobel 2003). In this context, the strong influence of sq1 likely reflects integrated effects of these edaphic properties on resource acquisition and biomass accumulation. In addition, oxygen availability to roots (sq4) also contributed substantially across species, particularly for *O. ficus‐indica* (13.1%) and *O. humifusa* (14.7%), suggesting that soil aeration and drainage are important constraints on distribution. Given the shallow and fibrous root systems characteristic of *Opuntia* (Cushman 2001; Nobel 2003), such constraints may directly influence establishment success under different soil conditions (Snyman 2006). Together, these findings indicate that climatic suitability alone does not fully account for invasion patterns in *Opuntia*. Instead, soil‐related factors interact with climatic conditions to shape distribution, highlighting the importance of incorporating edaphic variables to better characterize the realized niche and improve ecological inference in invasion studies (Beauregard and de Blois 2014; Behzadi et al. 2025).

### 4.3 Interspecific Variation Driven by Introduction History and Species‐Specific Traits

Within this multidimensional niche framework, the four *Opuntia* species exhibit marked interspecific variation in niche dynamics and geographic expansion. Among them, *O. ficus‐indica* shows the most extensive global distribution and the highest expansion ratio (46.2), despite relatively low niche overlap (Schoener's *D* = 0.284). This pattern likely reflects the combined effects of long‐term human‐mediated dispersal and intrinsic biological characteristics (Glon et al. 2020; Griffith 2004; Inglese et al. 2017). The species has been widely cultivated for food, forage, and dye production since pre‐Columbian times in Mesoamerica, and was subsequently introduced to Europe and Africa following the Columbian exchange (Inglese et al. 2017; Kiesling 1998). These repeated introductions across multiple continents have increased opportunities for establishment in environmentally suitable regions. Although representing the native niche of *O. ficus‐indica* with multiple wild ancestral taxa from central Mexico's arid uplands modestly broadens its baseline environmental space, relying on wild rather than cultivated material confirms that its broad invaded distribution is driven by environmental tracking across analogous climates, not simply an artifact of cultivation.

In addition to propagule pressure, biological traits may further facilitate its expansion (Tesfay et al. 2023). High genetic diversity reported in introduced populations (e.g., in Greece) suggests that multiple lineages contribute to invasive populations, potentially enhancing adaptive capacity and environmental tolerance (Ganopoulos et al. 2015; Smith et al. 2020). Moreover, experimental evidence indicates that spineless cultivars can rapidly revert to spiny wild forms within only a few generations after escaping cultivation, demonstrating strong phenotypic plasticity (Humphries et al. 2022; Novoa, Flepu, and Boatwright 2019). Such plastic responses may enable persistence across heterogeneous environments while remaining within a broadly conserved niche (Bates and Bertelsmeier 2021; Richards et al. 2006), thereby facilitating expansion without substantial niche shifts.

In contrast, the remaining species exhibit more constrained expansion patterns. *O. dillenii* shows a relatively high proportional expansion (expansion = 0.104) based on niche dynamics analysis, but its current distribution occupies only part of the predicted suitable area (Figure 6), suggesting incomplete range filling despite available suitable environments. Although all four species possess vertebrate‐dispersed fleshy fruits, published evidence does not suggest markedly lower dispersal capacity in *O. dillenii* relative to the other species (Padrón et al. 2011). Several factors may contribute to this incomplete range filling. As a relatively recent invader in parts of its introduced range, *O. dillenii* may not yet have reached distributional equilibrium, with suitable but as‐yet‐uncolonized areas reflecting ongoing dispersal lag (Václavík and Meentemeyer 2012). Biotic resistance, propagule pressure, and land‐use barriers in suitable but unoccupied areas may further constrain realized colonization (Liu et al. 2020). Consequently, incomplete range filling in *O. dillenii* is more likely to reflect constraints on spread and establishment following introduction, rather than intrinsic limitations in dispersal ability. *O. monacantha* exhibits expansion toward warmer and more humid environmental conditions in PCA‐env space, yet its distribution remains largely confined to environmentally analogous regions (MESS > 0), indicating expansion within, rather than beyond, the native environmental envelope. *O. humifusa* , by contrast, shows strong niche conservatism and limited expansion (expansion = 0.019; stability = 0.982), which may be linked to its distinct climatic constraints (Yan et al. 2024). Unlike most *Opuntia* species that are sensitive to frost damage, *O. humifusa* exhibits greater cold tolerance, enabling survival in temperate regions but simultaneously restricting its distribution to specific hydrothermal regimes (Goldstein and Nobel 1994; Leduc and Logan 2025; Loik and Nobel 1991). The marked ecological divergence among these morphologically similar congeners underscores the importance of accurate species‐level identification in distribution modeling, as misidentification or taxonomic lumping could substantially affect niche estimates and compromise the precision of early‐warning monitoring systems.

Taken together, these species‐specific patterns suggest that variation in expansion is not solely determined by environmental availability, but also by differences in dispersal history, realized occupancy, and physiological tolerance. Recognizing this interspecific variation is therefore essential for species‐level risk assessment and for designing targeted early‐warning systems in regions of projected climatic suitability. We further note that, for species with long cultivation histories, documented native ranges may include early‐naturalized areas near the center of origin, potentially inflating stability slightly; similarly, despite excluding records flagged as cultivated, some nonnative occurrences may still represent cultivated individuals, which could bias the estimated niche toward environments where the species is preferentially grown. However, because such residual records are expected to be few relative to the full occurrence dataset and contribute little weight to the availability‐corrected density estimates, such bias is unlikely to alter our qualitative conclusion of pervasive niche conservatism.

## 5 Conclusion

In summary, our results show that invasion in *Opuntia* species examined here is largely characterized by niche conservatism, with most species occupying environmental conditions analogous to their native ranges. Despite this overall pattern, interspecific differences in niche dynamics and geographic expansion indicate the role of species‐specific ecological constraints and introduction histories. Together, these findings highlight the interplay between niche conservatism and species‐specific variation in shaping plant invasion dynamics.

## Author Contributions

**Sifan Tao:** formal analysis (equal), investigation (equal), methodology (equal), visualization (equal), writing – original draft (equal), writing – review and editing (equal). **Xuefei Chen:** methodology (equal), resources (equal), writing – review and editing (equal). **Zhechen Qi:** conceptualization (equal), data curation (equal), funding acquisition (equal), investigation (equal), methodology (equal), project administration (equal), resources (equal), supervision (equal), validation (equal), writing – original draft (equal), writing – review and editing (equal). **Pan Li:** conceptualization (equal), data curation (equal), funding acquisition (equal), investigation (equal), methodology (equal), project administration (equal), resources (equal), supervision (equal), validation (equal), writing – original draft (equal), writing – review and editing (equal). **Xiaoling Yan:** conceptualization (equal), data curation (equal), funding acquisition (equal), investigation (equal), methodology (equal), project administration (equal), resources (equal), supervision (equal), validation (equal), writing – original draft (equal), writing – review and editing (equal).

## Funding

This study was funded by the Special Fund for Scientific Research of Shanghai Landscaping & City Appearance Administrative Bureau, grant numbers G252409 and G242412; Science and Technology Projects of Xizang Autonomous Region, grant numbers XZ202402ZD0005; the Natural Science Foundation of Zhejiang Province, grant number LY21C030008.

## Conflicts of Interest

The authors declare no conflicts of interest.

## Supporting information


**Table S1:** Occurrence records of the studied species.
**Table S2:** Environmental variables considered in this study.
**Table S3:** Performance evaluation of ten modeling algorithms for four Opuntia species under native and global datasets. Model performance was assessed using the area under the receiver operating characteristic curve (AUC), the true skill statistic (TSS), and Cohen's Kappa. Values are presented as mean ± SD from five‐fold cross‐validation. N (presences) and N (pseudo‐absences) indicate the number of occurrence records and pseudo‐absence points used in each model run, respectively.
**Table S4:** Loadings of eleven environmental variables on the first two principal component axes (PC1 and PC2) derived from PCA performed on the global background environmental space. Length represents the Euclidean distance of each variable vector in PC1–PC2 space.

## Data Availability

Occurrence records used in this study were obtained from the Global Biodiversity Information Facility (GBIF, https://www.gbif.org/). Bioclimatic and solar radiation data were sourced from the WorldClim database (https://www.worldclim.org/), and soil variables were obtained from the Harmonized World Soil Database (HWSD v1.2, https://gaez.fao.org/). The cleaned occurrence data after preprocessing are provided in the Supporting Information (Table S1). Data and scripts used for analysis are archived in Figshare (https://doi.org/10.6084/m9.figshare.32111794).
